# Running Water

**Published:** 1900-12-22

**Authors:** 


### RUNNING WATER.

Recent observations, made in various operation theatres, have tended strongly to confirm us in our love of running water and in our suspicion of basins. We cannot but feel that the basin and the method of washing which its use connotes are at the bottom of a good deal of the difficulty which some surgeons experience in attaining complete asepsis. Two factors are involved in almost all the processes adopted for the sterilisation of the hands. One is a matter of chemical disinfection, a microbicidal process by which the infectious microbes are destroyed; the other is a matter of mechanical removal. The latter process is really one of dilution, each successive water in which the hands are washed becoming charged with a smaller and smaller dose of the infection as the hands approach nearer and nearer to a condition of cleanliness. Even to the last, however, the hands can be no cleaner than the water by which they are being washed, and if this be borne in mind it becomes clear that frequent renewal of the water, or the solution used, must be far more important than the duration of the immersion, a matter on which so much stress is often laid. The ideal plan then is to wash in running water. One can hardly have a doubt that a far better effect is produced by the repeated application of a small quantity of clean antiseptic solution, followed each time by a swill under a running tap, than by even a prolonged soaking in a basin full of a solution which from the commencement to the end of the process is steadily becoming less and less charged with antiseptic, and more and more charged with infective material removed from the hands. Indeed, partly from a neutralisation of its chemical ingredients by the soap adherent to the hands, and partly from the growing charge of infection which it all the while receives, the trusted antiseptic may at the end of the appointed and orthodox time of immersion have become much more septic than the plain unsterilised water flowing from the tap. To wash the hands in repeated small quantities of a comparatively feeble but always clean antiseptic; to scrub between each application with a small quantity of water, not in a basin but flowing from a tap; and to wipe, when wiping is thought desirable, not on a large towel but upon a number of separate pieces of sterilised rag, each used but once, is likely to be attended with better results than a far more ostentatious scrubbing, a far more prolonged soaking, and the use of a far stronger solution, when each performance is done in a basin in the ordinary way.

